# The Glycemia Risk Index (GRI) as a Biomarker for Subclinical Endothelial Dysfunction in Type 1 Diabetes: A Cross-Sectional Study

**DOI:** 10.3390/ijms26189196

**Published:** 2025-09-20

**Authors:** Nicole Di Martino, Silvia Angelino, Antonietta Maio, Paolo Cirillo, Alessandro Pontillo, Mariangela Caputo, Lorenzo Scappaticcio, Paola Caruso, Miriam Longo, Giuseppe Bellastella, Maria Ida Maiorino, Katherine Esposito

**Affiliations:** 1 Unit of Endocrinology and Metabolic Diseases, University Hospital Luigi Vanvitelli, 80138 Naples, Italy; nicole.dimartino@unicampania.it (N.D.M.); silvia.angelino@unicampania.it (S.A.); paola.caruso@unicampania.it (P.C.); katherine.esposito@unicampania.it (K.E.); 2 Department of Advanced Medical and Surgical Sciences, University of Campania Luigi Vanvitelli, 80138 Naples, Italy; 3 Ph.D. Program in Translational Medicine, University of Campania Luigi Vanvitelli, 80138 Naples, Italy; 4 Department of Life Science, Health, and Health Professions, Link Campus University, 00165 Rome, Italy

**Keywords:** GRI, endothelial progenitor cells, type 1 diabetes, glucose control, glucose variability, endothelial dysfunction, cardiovascular risk

## Abstract

Circulating levels of endothelial progenitor cells (EPCs) involved in endothelial homeostasis are often reduced in people with type 1 diabetes (T1D). The Glycemia Risk Index (GRI) quantifies the quality of glucose control by assessing both hypo- and hyperglycemia risk. We aim to investigate the association between the GRI and circulating EPC levels in people with T1D. This cross-sectional study included 132 adults with T1D, on intensive insulin therapy. We calculated GRI from 14 days continuous glucose monitoring-derived metrics and quantified EPCs count by flow cytometry, stratifying results by GRI zones, ranging from A (lowest risk) to E (highest risk). Higher GRI scores were significantly associated with poorer metabolic parameters. Circulating levels of CD34^+^, CD133^+^, KDR^+^, and CD34^+^KDR^+^ cells were lower in participants with a worse GRI compared to adults with a better GRI. Linear regression analyses showed a negative association between GRI and CD34^+^ (β = −1.079, *p* = 0.006), CD34^+^CD133^+^ (β = −0.581, *p* = 0.008), and CD34^+^KDR^+^ (β = −0.147, *p* = 0.010). No significant association was found between HbA_1c_ and any EPC phenotype. Adults with T1D and a high GRI level had a lower EPCs count. GRI was significantly associated with certain EPC phenotypes, suggesting its potential role as a biomarker for cardiovascular risk assessment.

## 1. Introduction

Type 1 diabetes (T1D) is a chronic autoimmune disease characterized by pancreatic β-cell destruction, leading to severe insulin deficiency and chronic hyperglycemia [1]. Although traditionally considered a childhood disease, more than half of the type 1 diabetes cases are diagnosed in in adulthood, frequently with clinical features that may overlap with type 2 diabetes [2,3,4].

Maintaining optimal glycemic control remains a significant challenge in the management of T1D, as chronic hyperglycemia together with unstable glucose levels are associated with an increased risk of both micro- and macrovascular complications [5,6,7]. Indeed, despite significant advances in therapeutic strategies, people with type 1 diabetes (T1DM) still have a life expectancy that is about 13 years shorter than the general population [7,8,9,10,11]. Cardiovascular disease (CVD) is the leading cause of morbidity and mortality in individuals with T1D, remaining 4.2 times higher in people with T1DM compared with non-diabetic controls [6,12,13,14]. This excess risk is especially pronounced when the disease has an early onset, underscoring the cumulative burden of lifelong glycemic dysregulation on vascular health [12]. This challenge is particularly urgent given the rising incidence of type 1 diabetes [3]. One of the main contributors to CVD is the endothelial dysfunction induced by hyperglycemia [7,13,15,16].

Endothelial dysfunction results from the combination of multiple stressors, including chronic inflammation and increased oxidative stress. These factors lead to a reduced bioavailability of nitric oxide (NO), a key regulator of vascular homeostasis [17,18,19]. One of the mechanisms implicated in premature vascular aging is an impaired capacity for endothelial repair, for which circulating endothelial progenitor cells (EPCs) are critical mediators. EPCs are bone marrow-derived cells involved in maintaining endothelial homeostasis and repairing vascular damage [20]. Diabetes mellitus is characterized by defective mechanisms of vascular repair caused by an impaired regenerative capacity of EPCs [21]. Several studies have shown that circulating EPC levels are reduced in individuals with T1D [22,23]. In different studies, low EPCs count is associated with poor glycemic control, suggesting that both chronic hyperglycemia [23,24] and glucose variability [20,25,26] may directly impair endothelial regenerative capacity [23,24]. Moreover, a reduced EPC count has emerged as a powerful prognostic biomarker in high-risk populations, predicting future cardiovascular risk and mortality. Indeed, a meta-analysis of observational studies reported that a reduced baseline EPC level is associated with about a 2-fold increased risk of a combined cardiovascular end point and all-cause mortality [27].

Traditional measures of glycemic control, as glycated hemoglobin (HbA_1c_), reflect average glucose levels but do not capture short-term glucose fluctuations or glycemic variability, both of which are increasingly recognized as a key drivers of vascular damage through pathways involving oxidative stress and inflammation [28]. Recently, a novel composite metric derived from continuous glucose monitoring (CGM) data has been introduced, named the Glycemia Risk Index (GRI) [26]. This metric simultaneously quantifies the risk of hypo- and hyperglycemia, providing a comprehensive assessment of glycemic control quality. This index can be categorized and graphically represented across five zones (quintiles), labeled A to E, corresponding to the best (1st–20th percentile) to worst (81st–100th percentile) quality of glycemic control [26]. There is evidence from observational studies that high GRI values are associated with increased risks of both diabetic microvascular and macrovascular complications [29,30].

While it is established that both chronic hyperglycemia and glucose variability can impair EPC number and function, traditional metrics like HbA_1c_ fail to capture the full spectrum of glycemic dysregulation, particularly the combined risk of hypo- and hyperglycemia. The GRI offers a more holistic assessment of these glucose fluctuations; however, it remains unknown whether this comprehensive metric is associated with biological markers of vascular repair, such as EPCs, representing a critical research gap. Therefore, this study was designed to be the first to (1) characterize the distribution of circulating EPCs across GRI zones and (2) investigate the association between circulating EPC levels and glycemic control, assessed by the GRI in adults with type 1 diabetes. This will help elucidate the mechanisms linking unstable glycemic control with the heightened risk of vascular complications in young adults with type 1 diabetes.

## 2. Results

### 2.1. Demographic Analysis

A total of one-hundred and thirty-two adults with T1DM met the eligibility criteria and were included in the study. The clinical and demographic characteristics of the study population are illustrated in Table 1. Seventy-four (56.1%) participants were females, and forty-six (34.8%) were smokers. The median age was 24.5 years, and the median diabetes duration was 16.0 years. Median fasting plasma glucose was 180.0 mg/dL and median HbA_1c_ was 7.4%. Ninety participants (68.2%) were treated with CSII, with a median total daily insulin dose of 46.5 UI/day. Nine patients (7%) had microvascular complications (four diabetic sensorimotor polyneuropathy, four diabetic non-proliferative retinopathy, and one diabetic kidney disease stage III).

Participants were included into five glycemic risk zones according to GRI: zone A (N = 10), zone B (N = 34), zone C (N = 48), zone D (N = 26), and zone E (N = 14). The clinical variables of the study population stratified by GRI zones are depicted in Table 2. The median age was not different among the five groups, nor were diabetes duration, blood pressure, HDL cholesterol, and triglycerides. Moreover, participants with the worst GRI, zones C–E, showed fasting plasma glucose values significantly higher than those with a better GRI [zone A vs. zones C–E, mean (SD), mg/dL, 93.6 (47.1) vs. 188.7 (72.5) vs. 170.3 (58.3) vs. 225.6 (98.3), *p* < 0.001], as HbA_1c_ [zone A vs. zones C–E, median (IQR), %, 6.8 (6.4–6.8) vs. 7.5 (7.2–8.2) vs. 8.0 (7.2–8.2) vs. 8.3 (6.8–11.8), *p* < 0.001]. Differences were also observed in the lipid profile, which was significantly worse in participants exhibiting GRI values in unfavorable zones. Higher levels of total cholesterol were observed in participants in zone C compared to those in zone B [zone B vs. zone C, median (IQR), 153.0 (142.0–176.0) vs. 179.0 (160.0–191.5), *p* = 0.040]. Participants in zone E showed higher LDL-cholesterol levels than those in zone B [zone B vs. zone E, median (IQR), 86.0 (62.2–98.4) vs. 101.0 (90.0–118.0), *p* = 0.039], and participants in zone C had higher triglycerides levels than zona A [zone A vs. zone C, median (IQR), 55.0 (50.0–58.0) vs. 80.5 (62.0–86.0), *p* = 0.026], while HDL-cholesterol levels showed no significant differences between the five groups (*p* = 0.776).

### 2.2. Descriptive Analysis of Circulating Endothelial Progenitor Cells

**Figure 1:**
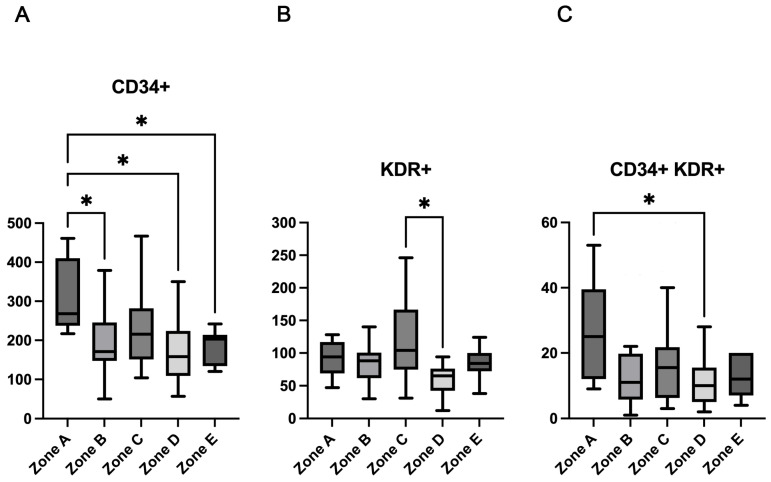
CD34^+^, KDR^+^, and CD34^+^KDR^+^ cell count across GRI zones. Box plots illustrate the median and interquartile range for the EPC phenotypes that showed significant differences among groups: (**A**) CD34^+^ cells, (**B**) KDR^+^ cells, and (**C**) CD34^+^KDR^+^ cells. The following symbol * indicates statistically significant differences in post-hoc comparisons, as determined by Dunn’s test. * *p* < 0.05.

Table 3 shows the count of the different EPCs phenotypes according to the five GRI zones. Although a consistent linear decrease was not observed, a general pattern emerged where EPC counts were lower in zones representing poorer glycemic quality. Specifically, participants in zones B, D, and E had significantly lower counts of CD34^+^ cells compared to zone A (*p* < 0.001) (Figure 1A). While a significant overall difference was detected among the groups for the CD133^+^ EPC phenotype (*p* = 0.049), the subsequent post-hoc analysis did not reveal statistically significant differences between any two individual zones. KDR^+^ cells were significantly lower in zone D as compared with those in zone C (Figure 1B). There was a significant difference in circulating levels of CD34^+^KDR^+^ cells, which were significantly lower in participants of zone D as compared with those in zone A (Figure 1C). There were no significant differences in median values of CD133^+^KDR^+^ and CD34^+^CD133^+^KDR^+^ cells across the GRI zones.

### 2.3. Regression Analyses Results

Results from linear regression analyses between EPCs phenotypes and GRI are reported in Table 4 and Table S1. In the overall study population, higher GRI values resulted as an independent predictor for decreasing levels of CD34^+^ (β-coefficient = −1.079, *p* = 0.006), CD34^+^CD133^+^ (β-coefficient = −0–581, *p* = 0.008), and CD34^+^KDR^+^ (β-coefficient = −0.147, *p* = 0.010) cell count. These associations remained significant after adjusting for confounders (Table S2). In contrast, no significant association was observed between GRI and CD133^+^, KDR^+^, CD133^+^KDR^+^, and CD34^+^CD133^+^ KDR^+^ cells.

Additional linear regression analyses were performed to investigate the correlation between circulating EPCs and HbA_1c_ (Table 5 and Table S3). These analyses suggested that there was no significant association between the EPC phenotypes and HbA_1c_, even after adjusting for confounders (Table S4).

## 3. Discussion

To the best of our knowledge, this is the first study that evaluated the association between GRI and circulating EPC levels in a cohort of adults with type 1 diabetes. We found that higher GRI values, which reflect poor and unstable glycemic control, were associated with lower counts of specific EPC phenotypes, particularly CD34^+^, CD34^+^CD133^+^, and CD34^+^KDR^+^ cells, whereas HbA_1c_ showed no association with any EPC phenotype. These findings suggest a potential mechanism of impaired vascular repair induced by glucose variability that is not captured by HbA_1c_.

Our study, which focused on a cohort of young adults with type 1 diabetes who had fair glyco-metabolic control and were generally free of diabetic complications, revealed that participants with a worse glycemic risk, as indicated by a higher GRI, also had higher HbA_1c_ and glucose variability (CV) levels, alongside reduced circulating levels of different phenotypes of EPCs. These results are consistent with those emerging by other literary studies, which have demonstrated that people with T1D often exhibit a decrease in both the number and function of EPCs, key cellular biomarkers of cardiovascular health [31]. More importantly, our findings support the growing body of evidence suggesting that this decline in regenerative cells is more strongly associated with glycemic variability rather than with mean chronic hyperglycemia alone [32,33]. GRI zones may detect a gradual increase in pathophysiological stress induced by glucose variability. Zone A, the ‘very low risk’ zone, likely reflects a stable glycemic environment, which may be optimal for EPC survival and function. Moving towards the ‘very high risk’ zone (E), the exposure to marked glycemic variability increases, with frequent hyperglycemic and hypoglycemic events. Such an environment could be toxic for EPCs. The oxidative stress induced by hyperglycemic fluctuations, along with the systemic inflammation triggered by hypoglycemia, may lead to EPC apoptosis and dysfunction. Therefore, the significant decrease in CD34^+^, KDR^+^, and CD34^+^KDR^+^ phenotypes observed in participants from high GRI zones, compared to those in zones with better glycemic control, could be a direct biological consequence of the prolonged exposure to this adverse glycemic environment. This suggests progressive failure of the vascular repair system under the burden of unstable glycemic control.

Of note, different studies showed that oscillating glucose is more deleterious to endothelial function and induces greater oxidative stress than sustained high glucose [32]. In addition, persistent glucose variability may result in the occurrence of excessive glycemic excursions, which, in turn, have been shown to increase the risk of developing hyperglycemia or hypoglycemia [32]. Therefore, these excessive glycemic excursions likely play a critical role in impairing the vascular repair mechanisms in young individuals with T1D.

Previous studies suggest that in children and young adults with T1D, poor glycemic control, defined by the worsening of HbA_1c_ levels over time [34] or high glucose variability [35], correlates with a marked depletion of circulating EPCs. Our findings align with and expand upon this, demonstrating that poorer quality of glucose control, as expressed by high GRI values, corresponds to a lower EPC count. A key finding of our study, however, is that HbA_1c_ was found to be a less effective predictor of EPC circulating levels compared to the GRI. While HbA_1c_ is the established gold standard for the evaluation of long-term glucose control, our results suggest it may have some limitations in predicting the early stage of endothelial damage. On the other hand, EPCs levels strongly correlated with GRI, highlighting the importance of composite metrics that capture both hyper- and hypoglycemic excursions. The inclusion of time spent in hypoglycemia in the GRI formula is relevant, as hypoglycemic events are themselves linked to cardiovascular disease and mortality. By encompassing the full spectrum of glycemic variability, the GRI may therefore provide a more comprehensive assessment of both glucose control and risk of microvascular complications, even in their earlier phases of development [36,37].

We found that higher GRI values are associated with a reduction in CD34^+^, CD34^+^CD133^+^, and CD34^+^KDR^+^ cells. The markers CD34^+^ and CD133^+^ are typically associated with hematopoietic progenitor cells, representing a more immature, uncommitted cell population. On the other hand, the CD34^+^KDR^+^ phenotype identifies a population of progenitor cells that not only possesses stem-like qualities (CD34^+^) but has also begun differentiating into endothelial cells (KDR^+^). This impairment in the availability of “endothelial-committed” progenitors could be a key mechanism linking unstable glycemic control to the pathogenesis of vascular complications in type 1 diabetes. This is relevant as young adults with type 1 diabetes are often free from overt complications; therefore, a depletion of progenitor cells may represent a critical, subclinical sign of impaired vascular repair capacity. Identifying a reduction in this “endothelial-committed” cell pool (CD34^+^KDR^+^) early in the disease course may signal the onset of premature vascular aging long before complications become clinically apparent. In observational studies of people with type 2 diabetes, the count of CD34^+^KDR^+^ cells negatively correlated with the severity of diabetic complications [38], meaning fewer cells were associated with more severe disease. Whether this applies also to type 1 diabetes needs to be further investigated. However, one study reported that continuous subcutaneous insulin infusion in T1D patients improved levels of CD34^+^KDR^+^ cells more effectively than multiple daily injections, linking the improvement to lower glucose variability [25].

The link between poor glycemic control, as holistically captured by the GRI, and the EPCs depletion may be explained by several biological mechanisms. The glycemic variability and frequent excursions into hyper- and hypoglycemia that result in a high GRI are known to induce profound oxidative stress and promote the formation of advanced glycation end-products (AGEs) [18,32]. These pathological conditions are known to directly impair EPC function and mobilization from the bone marrow [39]. Mechanistically, this occurs through the downregulation of critical signaling pathways like VEGF/PI3K/Akt/eNOS and the reduced bioavailability of nitric oxide (NO) and stromal cell-derived factor-1 (SDF-1), a key chemokine for EPC homing to sites of injury [40,41].

The present study has limitations. The cross-sectional design does not allow for making any inferences regarding cause and effect. It is therefore not possible to determine whether higher glycemic variability, reflected by the GRI, leads to a reduction in EPCs, or if a pre-existing deficiency in vascular repair capacity contributes to glycemic instability. While the used single-center convenience sampling method limits the generalizability of our findings, the cohort is considered representative of young adults with T1D under intensive insulin therapy and with relatively fair metabolic control, a key demographic for studying the early stages of endothelial dysfunction. Further studies should be focused on broader populations, such as older individuals, those with a longer duration of diabetes, or patients with more advanced complications. This study has also several key strengths. This is the first study to investigate the relationship between the GRI and circulating EPCs in adults with T1D. This provides new insights into how a comprehensive metric of glycemic control relates to a crucial biomarker of vascular health. The study utilizes the GRI, a modern and robust metric derived from continuous glucose monitoring (CGM) data. The GRI’s ability to simultaneously quantify the risk of both hypo- and hyperglycemia provides a more holistic view of glycemic control than traditional measures like HbA_1c_. The finding that GRI is a significant predictor of EPC levels while HbA_1c_ is not highlights the importance of CGM-derived metrics for assessing the risk of endothelial damage. This suggests that the GRI may serve as an earlier indicator of endothelial dysfunction and microvascular complications than HbA_1c_. This study focuses on a cohort of young adults with T1D who are largely free of significant complications. This allows for the investigation of early signs of vascular damage, providing valuable information about the initial stages of diabetes-related complications.

## 4. Materials and Methods

### 4.1. Study Population

This study was conducted according to the Strengthening the Reporting of Observational Studies in Epidemiology (STROBE) Statement: guidelines for reporting observational studies (Supplementary Material) [42].

This is an observational cross-sectional study that consecutively involved subjects with type 1 diabetes followed at the Diabetes Unit of the University Hospital of Campania “Luigi Vanvitelli” in Naples (Italy) from January 2025 to May 2025. We included men and women with type 1 diabetes, aged > 18 years, who were receiving intensive insulin treatment in conjunction with CGM and in whom the percentage of CGM sensor use was >70% during the time of observation. Exclusion criteria were (1) pregnancy; (2) presence of acute diabetes-related disorders (acute hypoglycemia, diabetic ketoacidosis); (3) acute illness or concomitant chronic diseases (assessed with anamnesis and laboratory tests) that may favor glucose variability; and (4) use of drugs or any kind of substances able to interfere with glucose levels. To prevent selection bias, all eligible individuals who presented to our clinic during the recruitment period were enrolled consecutively, irrespective of their sex or other demographic characteristics. The resulting cohort is considered reflective of the sex distribution typically observed in the population of young adults with type 1 diabetes managed at our tertiary care center. All participants provided written informed consent, as approved by our Institutional Review Board, which covered the collection and use of their anonymized clinical and demographic data for research purposes.

### 4.2. Clinical Variables

Clinical data were collected from electronic medical records and transferred into an internal clinical database. Age, sex, weight, height, body mass index (BMI, calculated as weight in kilograms divided by the square of height in meters), duration of diabetes, smoking habits (defined as habitual active smoking of one or more cigarettes per day), blood pressure (BP), fasting plasma glucose (FPG), glycated hemoglobin A1c (HbA_1c_), total cholesterol, low-density (LDL) and high-density lipoprotein (HDL) cholesterol, triglyceride levels, other autoimmunity diseases, and the presence of micro and macrovascular complications were collected for each participant.

CGM-related metrics of the 14 days before the visit were collected using commercially available systems [including Dexcom G6 (Dexcom, Inc., San Diego, CA, USA), Medtronic Guardian Sensor 4 (Medtronic, Minneapolis, MN, USA), and Abbott FreeStyle Libre 3 (Abbott Diabetes Care, Alameda, CA, USA)] from their respective web-based platforms (e.g., Dexcom Clarity (Dexcom, Inc., San Diego, CA, USA), CareLink (Medtronic, Northridge, CA, USA), and LibreView Abbott Diabetes Care, Alameda, CA, USA)] and analyzed by displaying the ambulatory glucose profile (AGP). They included the coefficient of variation (CV), the Glucose Management Indicator (GMI), the mean glucose, the percentage of time spent in the range of normoglycemia (TIR, 70–180 mg/dL), the percentage of time spent below range (TBR) level 1 (70–55 mg/dL) and level 2 (<55 mg/dL), and the percentage of time spent above range (TAR) level 1 (180–250 mg/dL) and level 2 (>250 mg/dL). The GMI was calculated automatically by the appropriate system (Dexcom, Medtronic or Abbott). The Glycemia Risk Index (GRI) was computed integrating data related to the proportion of time spent in specific glycemic intervals, based on standardized metrics of the AGP. These include the following:

- VLow (very low glucose hypoglycemia): Time spent at glucose levels <54 mg/dL;
- Low (low glucose hypoglycemia): Time spent at glucose levels 54 to <70 mg/dL;
- High (high glucose hyperglycemia): Time spent at glucose levels 180 to 250 mg/dL;
- VHigh (very high glucose hyperglycemia): Time spent at glucose levels >250 mg/dL.

The Glycemia Risk Index (GRI) was calculated using the following equation: GRI = (3.0 * VLow) + (2.4 * Low) + (1.6 * VHigh) + (0.8 * High) [26]. This formula gives greater weight to episodes of hypoglycemia and extreme hyperglycemia, thus more accurately reflecting the risks associated with these events.

### 4.3. Assessment of Circulating Levels of EPCs

We identified seven EPC phenotypes to comprehensively characterize the circulating progenitor cell pool, based on the combination of three key surface antigens: CD34 and CD133, which denote a hematopoietic stem/progenitor origin, and KDR (VEGFR-2), which indicates commitment to the endothelial lineage.

Assessment of circulating levels of EPCs was performed on fresh blood samples collected in citrate tubes after overnight fasting. Peripheral blood cells were analyzed for the expression of the above-mentioned surface antigens by direct flow cytometry, as previously described [25]. Mononuclear cells were isolated from peripheral venous blood by density centrifugation. Then, the isolated blood cells were stained for 30 min at 4 °C in the dark with fluorescein isothiocyanate (FITC)-conjugated anti-human CD34 monoclonal antibody (mAb) (E-AB-F1143C, Elabscience, Wuhan, China), phycoerythrin (PE)-conjugated antihuman KDR mAb (BD Biosciences, Franklin Lakes, NJ, USA, Cat# 560494, RRID:AB_1645503, https://www.antibodyregistry.org/AB_1645503, accessed on 11 September 2025), and allophycocyanin (APC)-conjugated antihuman CD133 (Miltenyi Biotec, San Jose, CA, USA, Cat# 130-113-106, RRID:AB_2725935, https://www.antibodyregistry.org/AB_2725935, accessed on 11 September 2025). Isotope immunoglobulin IgG1 and IgG2a antibody was used to discriminate between signal range and baseline fluorescence within the samples. Spectral overlap compensation was performed manually using the CellQuest Pro software (version 5.1). To this end, single-stained compensation controls (using either cells or beads) for FITC, PE, and APC were acquired. A compensation matrix was then created by manually adjusting the percentage of spectral overlap subtraction for each fluorochrome. The correction was considered optimal when the median fluorescence intensity of the positive population in the spillover channel aligned with that of the negative population. This manually validated compensation matrix was then applied to all experimental samples during analysis. The gating strategy was established using a combination of controls: (1) unstained cells to define the baseline autofluorescence, (2) isotype-matched immunoglobulin controls (IgG1 and IgG2a) to account for non-specific antibody binding, and (3) fluorescence minus one (FMO) controls for each fluorochrome to accurately set the gates for positive populations, particularly with regard to KDR expression in the context of CD34 and CD133 co-expression. After incubation, quantitative analysis was performed on a BD FACSCalibur flow cytometer (Becton Dickinson, San Jose, CA, USA), and 1,000,000 cells were acquired in each sample. A morphological gate was used to exclude granulocytes (Figure S1A). Then, we gated CD34^+^ (Figure S1B) or CD133^+^ (Figure S1C) peripheral blood cells in the lymphocytes cell fraction and examined the resulting population for the dual expression of KDR to detect the CD34^+^KDR^+^ (Figure S1D) and the CD133^+^KDR^+^ cells (Figure S1E). Total KDR^+^ mononuclear cells were identified separately (Figure S1F). In the two-dimensional dotplot analysis, we identified CD34^+^CD133^+^ cells (Figure S1G). Triple-positive cells were identified by the dual expression of KDR and CD133 in the CD34^+^ gate (Figure S1H). Data were processed with the use of the Macintosh CELLQuest software (version 3.5.1) program (Becton Dickinson). Measures were repeated twice in two separate blood samples. The instrument setup was optimized daily by analyzing the expression of peripheral blood lymphocytes labeled with an anti-CD4 FITC/CD8 PE/CD3 PECy5/CD45APC 4-color combination. The results from flow cytometry were expressed as the number of cells per 10^6^ events.

### 4.4. Statistical Analysis

An a priori sample size calculation was not performed, as this was the first study to investigate the association between the GRI and circulating EPC levels, meaning no prior data were available for effect size estimation. Therefore, the sample size was determined by the consecutive enrollment of all eligible individuals who attended our center in the above-mentioned timeframe. This approach yielded a cohort of 132 participants. The primary outcome was to analyze the distribution of EPC counts according to the GRI zones in the study population; the secondary outcome was to investigate the association between GRI and EPC levels. To confirm the statistical adequacy of our sample size, a post hoc power analysis was conducted on our primary outcome. The analysis of the overall comparison among groups (one-way ANOVA), considering a sample size of 132 subjects, a mean value of 13.7, and a standard deviation of 11.24, showed a power of 0.81. This suggests that the study had sufficient power to identify overall group differences while maintaining an acceptable level of risk for type II error.

Descriptive statistics were used to characterize the demographic and clinical characteristics of all participants in the study sample. The Kolmogorov–Smirnov test was used to analyze the variables distribution. Based on this analysis, continuous variables which were normally distributed were presented as mean and standard deviation (SD), while non-normally distributed variables were presented as median and interquartile range (IQR) [43]. Categorical variables were presented as frequencies and percentages. Analysis of variance, one-way ANOVA for normally distributed variables, or ANOVA on ranks for non-normally distributed variables, followed by Bonferroni or Dunn’s post hoc test, was performed to assess the differences of continuous variables between groups. Linear regression analysis was used to test the associations of circulating EPCs levels and GRI or HbA_1c_, with the sign indicating the direction of the correlation. The regression models were adjusted for potential confounders, including age, diabetes duration, and body mass index. A *p* value lower than 0.05 was considered statistically significant. All statistical analyses were performed using SPSS software (version 27.0, SPSS, Chicago, IL, USA).

## 5. Conclusions

In conclusion, this is the first study to demonstrate that young adults with T1D and higher GRI, reflecting poorer and more variable glycemic control, exhibited lower counts of EPCs—particularly the CD34^+^, CD34^+^CD133^+^, and CD34^+^KDR^+^ phenotypes. The significant inverse association between the GRI and the levels of EPCs may suggest a role for GRI as a clinical tool to identify patients with a higher risk for endothelial dysfunction and vascular diabetic complications. Further multi-center studies, with prospective design, should confirm these findings and clarify the predictive role of the GRI in the long-term development of diabetes-related vascular complications.

## Figures and Tables

**Table 1:** Clinical and demographic characteristics of participants in the study.

Variables	Study Cohort (n = 132)
Demographics	
Age, years	24.5 (23.0–29.0)
Diabetes duration, years	16.0 (12.0–22.0)
Female, n (%)	74 (56.1)
Smokers, n (%)	46 (34.8)
Body weight, kg	67.0 (60.7–76.0)
BMI, kg/m^2^	23.7 (21.9–26.2)
SBP, mmHg	110.0 (105.0–120.0)
DBP, mmHg	70.0 (65.0–80.0)
Glycemic markers	
Fasting plasma glucose, mg/dL	180.0 (108.0–212.0)
HbA_1c_, %	7.4 (6.8–8.2)
GRI, %	48.3 (36.8–66.2)
TIR, %	59.5 (46.0–69.0)
CV, %	37.2 (33.5–41.2)
Mean Glucose, mg/dL	168.5 (151.0–184.0)
GMI, %	7.3 (6.9–7.8)
Lipid profile	
Total Cholesterol, mg/dL	167.5 (147.0–185.0)
HDL Cholesterol, mg/dL	59.5 (50.0–70.0)
LDL Cholesterol, mg/dL	91.0 (74.0–106.8)
Triglycerides, mg/dL	69.0 (52.0–85.0)
Insulin therapy	
Total daily insulin dose, UI/die	46.5 (37.7–55.7)
Basal daily insulin dose, UI/die	24.0 (19.5–28.0)
Bolus daily insulin dose, UI/die	21.0 (18.0–26.8)
Comorbidities	
Other autoimmune diseases, n (%)	40 (30.3)
Other therapies, n (%)	42 (31.8)
CSII users, n (%)	90 (68.2)

As for sample distribution, continuous variables are expressed in median and interquartile range (IQR), and categorical variables with numbers and percentages. Abbreviations: BMI, body mass index; CSII, continuous subcutaneous insulin infusion; CV, coefficient of variation; DBP, diastolic blood pressure; GMI, glucose monitoring indicator; HbA_1c_, Hemoglobin A1c; HDL cholesterol, high-density lipoprotein cholesterol; LDL cholesterol, low-density lipoprotein cholesterol; SBP, systolic blood pressure; TIR, time in range.

**Table 2:** Clinical variables in the study population stratified by GRI zones.

Variables	Zone A (n = 10)	Zone B (n = 34)	Zone C (n = 48)	Zone D (n = 26)	Zone E (n = 14)	*p*
Age, years	33.0 (23.0–33.0)	24.0 (22.0–31.0)	24.5 (23.0–29.0)	25.0 (22.0–26.0)	24.0 (23.0–27.0)	0.349
Diabetes duration, years	22.0 (10.0–35.0)	13.0 (12.0–20.0)	16.0 (12.0–22.0)	16.0 (14.0–22.0)	18.0 (7.0–19.0)	0.714
Body weight, kg	62.0 (56.4–63.0) **	71.0 (62.0–77.0)	64.0 (59.6–73.0) **	76.0 (65.5–81.2)	65.0 (60.7–67.0) **	<0.01
BMI, kg/m^2^	21.0 (20.5–23.6)	24.9 (22.9–27.8)	23.3 (20.9–24.0)	26.5 (21.3–29.1) *	24.1 (22.5–25.5)	<0.01
SBP, mmHg	110 (110–120)	110 (105–120)	117.5 (110–122.5)	120 (100–120)	105 (100–110)	0.114
DBP, mmHg	80 (75–80)	70 (65–75)	72.5 (70–80)	70 (65–80)	70 (60–75)	0.021
Fasting plasma glucose, mg/dL	93.6 ± 47.1	150.2 ± 62.3	188.7 ± 72.5 *	170.3 ± 58.3 *	225.6 ± 98.3 *^§^	<0.01
HbA_1c_, %	6.8 (6.4–6.8)	7.2 (6.5–8.0)	7.5 (7.2–8.2) *	8.0 (7.2–8.2) *	8.3 (6.8–11.8) *	<0.01
Total cholesterol, mg/dL	165.0 (146.0 182.0)	153.0 (142.0–176.0)	179.0 (160.0–191.5) ^§^	169.0 (153.0–175.0)	177.0 (147.0–192.0)	0.040
LDL cholesterol, mg/dL	84.0 (74.0–105.0)	86.0 (62.2–98.4)	95.2 (75.2–111.5)	79.0 (74.4–104.4)	101.0 (90.0–118.0) ^§^	0.039
HDL cholesterol, mg/dL	65.0 (58.0–68.0)	56.0 (48.0–71.0)	59.0 (50.0–67.5)	62.0 (59.0–67.0)	70.0 (36.0–77.0)	0.776
Triglycerides, mg/dL	55.0 (50.0–58.0)	66.0 (49.0–80.0)	80.5 (62.0–86.0) *	76.0 (51.0–88.0)	61.0 (57.0–94.0)	0.026
TIR, %	86.0 (79.0–86.0)	70.0 (68.0–76.0)	58.0 (52.0–63.5)	45.0 (37.0–47.0)	32.0 (28.0–37.0)	<0.01
CV, %	29.3 (28.8–30.0)	33.5 (32.0–37.4)	39.7 (35.3–42.7) *^§^	44.0 (35.8–45.1) *^§^	36.1 (35.0–41.2) *	<0.01
Mean glucose, mg/dL	147.0 (124.0–183.0)	152.0 (149.0–161.0)	168.5 (153.0–179.5)	179.0 (168.0–185.0) ^§^	210.0 (189.0–235.0) *^§^	<0.01
GMI, %	6.4 (6.3–6.8)	6.9 (6.8–7.2)	7.4 (7.0–7.8) *^§^	7.7 (7.6–8.1) *^§^	8.7 (8.1–9.6) *^§^	<0.001

As for sample distribution, continuous variables are expressed in mean and standard deviation (SD) or median and interquartile range (IQR). Abbreviations: BMI, body mass index; CV, coefficient of variation; DBP, diastolic blood pressure; GMI, glucose monitoring indicator; HbA_1c_, hemoglobin A1c; HDL cholesterol, high-density lipoprotein cholesterol; LDL cholesterol, low-density lipoprotein cholesterol; SBP, systolic blood pressure; TIR, time in range. * *p* < 0.05 vs. zone A; ** *p* < 0.05 vs. zone D; ^§^ *p* < 0.05 vs. zone B.

**Table 3:** EPCs count in the study population stratified by GRI zones.

Phenotypes	Zone A (n = 10)	Zone B (n = 34)	Zone C (n = 48)	Zone D (n = 26)	Zone E (n = 14)	*p*
CD34^+^	268.0 (244.0–393.0)	171.0 (148.0–226.0) *	215.0 (155.0–274.5)	158.0 (113.0–215.0) *	203.0 (134.0–214.0) *	<0.001
CD133^+^	239.0 (226.0–266.0)	159.0 (134.0–239.0)	201.5 (162.5–245.5)	159.0 (133.0–204.0)	198.0 (164.0–358.0)	0.049
KDR^+^	94.0 (76.0–113.0)	88.0 (63.0–99.0)	103.0 (78.0–166.0)	65.0 (45.0–71.0) ^¶^	84.0 (72.0–100.0)	<0.001
CD34^+^CD133^+^	88.0 (66.0–166.0)	80.0 (54.0–116.0)	80.5 (58.0–101.5)	80.0 (65.0–106.0)	70.0 (17.0–110.0)	0.654
CD34^+^KDR^+^	25.0 (13.0–35.0)	11.0 (6.0–19.0)	15.5 (6.5–21.5)	10.0 (5.0–15.0) *	12 (7.0–20.0)	0.034
CD133^+^KDR^+^	3.0 (3.0–5.0)	4.0 (2.0–6.0)	5.5 (2.0–8.0)	4.0 (3.0–6.0)	6.0 (2.0–10.0)	0.511
CD34^+^CD133^+^ KDR^+^	2.0 (1.0–2.0)	2.0 (1.0–3.0)	2.0 (1.0–4.0)	2.0 (1.0–4.0)	3.0 (1.0–5.0)	0.651

As for sample distribution, continuous variables are expressed in median and interquartile range (IQR). EPCs count is expressed as number/10^6^ events. The *p*-value represents the overall significance across the five groups, calculated using the Kruskal–Wallis test. Symbols indicate significant differences in post-hoc pairwise comparisons (Dunn’s test): * *p* < 0.05 vs. A; ^¶^ *p* < 0.05 vs. C.

**Table 4:** Linear regression analysis between different EPC phenotypes levels (dependent variable) and GRI (independent variable).

	β	*p*	R^2^
CD34^+^	−1.079	0.006	0.06
CD133^+^	−0.426	0.233	0.01
KDR^+^	−0.481	0.089	0.02
CD34^+^CD133^+^	−0.581	0.008	0.06
CD34^+^KDR^+^	−0.147	0.010	0.05
CD133^+^KDR^+^	0.020	0.209	0.01
CD34^+^CD133^+^KDR^+^	0.007	0.437	0.01

**Table 5:** Linear regression analysis between different EPC phenotypes levels (dependent variable) and HbA_1c_ (independent variable).

	β	*p*	R^2^
CD34^+^	−10.096	0.123	0.02
CD133^+^	6.572	0.268	0.01
KDR^+^	−5.326	0.259	0.01
CD34^+^CD133^+^	−0.889	0.809	0.0
CD34^+^KDR^+^	−1.315	0.170	0.01
CD133^+^KDR^+^	−0.185	0.482	0.004
CD34^+^CD133^+^KDR^+^	−0.151	0.304	0.01

## Data Availability

The original contributions presented in this study are included in the article/Supplementary Material. Further inquiries can be directed to the corresponding author.

## Supplementary Materials

The following supporting information can be downloaded at: https://www.mdpi.com/article/10.3390/ijms26189196/s1.

## Abbreviations

The following abbreviations are used in this manuscript:

EPCs	Endothelial Progenitor Cells
GRI	Glycemia Risk Index
T1D	Type 1 Diabetes
CGM	Continuous Glucose Monitoring
