# Supplementary material for: The Glycemia Risk Index (GRI) as a Biomarker for Subclinical Endothelial Dysfunction in Type 1 Diabetes: A Cross-Sectional Study
Source: Int J Mol Sci. 2025 Sep 20;26(18):9196. doi: 10.3390/ijms26189196 (PMC12470873; doi:10.3390/ijms26189196)
Supplement: Supplementary file 1 [file ijms-26-09196-s001.zip › STROBE Statement.pdf]

STROBE Statement—Checklist of items that should be included in reports of ***cross-sectional studies***

	Item No	Recommendation	Pag
**Title and abstract**	1	(*a*) Indicate the study's design with a commonly used term in the title or the abstract	1, title page
		(*b*) Provide in the abstract an informative and balanced summary of what was done and what was found	1, abstract
**Introduction**			
Background/rationale	2	Explain the scientific background and rationale for the investigation being reported	1,2, introduction
Objectives	3	State specific objectives, including any prespecified hypotheses	1,2, introduction
**Methods**			
Study design	4	Present key elements of study design early in the paper	8, Materials and Methods (study population)
Setting	5	Describe the setting, locations, and relevant dates, including periods of recruitment, exposure, follow-up, and data collection	8-10, Materials and Methods
Participants	6	(*a*) Give the eligibility criteria, and the sources and methods of selection of participants	8, Materials and Methods (study population)
Variables	7	Clearly define all outcomes, exposures, predictors, potential confounders, and effect modifiers. Give diagnostic criteria, if applicable	9-10, Materials and Methods
Data sources / measurement	8*	For each variable of interest, give sources of data and details of methods of assessment (measurement). Describe comparability of assessment methods if there is more than one group	10, Materials and Methods (statistical analysis)
Bias	9	Describe any efforts to address potential sources of bias	10, Materials

			and Methods (statistical analysis)
Study size	10	Explain how the study size was arrived at	10, Materials and Methods (statistical analysis)
Quantitative variables	11	Explain how quantitative variables were handled in the analyses. If applicable, describe which groupings were chosen and why	10, Materials and Methods (statistical analysis)
Statistical methods	12	(*a*) Describe all statistical methods, including those used to control for confounding	10, Materials and Methods (statistical analysis)
		(*b*) Describe any methods used to examine subgroups and interactions	10, Materials and Methods (statistical analysis)
		(*c*) Explain how missing data were addressed	NA
		(*d*) If applicable, describe analytical methods taking account of sampling strategy	NA
		(*e*) Describe any sensitivity analyses	NA
**Results**			
Participants	13*	(a) Report numbers of individuals at each stage of study—eg numbers potentially eligible, examined for eligibility, confirmed eligible, included in the study, completing follow-up, and analysed	3, results
		(b) Give reasons for non-participation at each stage	-
		(c) Consider use of a flow diagram	-
Descriptive data	14*	(a) Give characteristics of study participants (eg demographic, clinical, social) and information on exposures and potential confounders	3,4, Table 1, results
		(b) Indicate number of participants with missing data for each variable of interest	NA
Outcome data	15*	Report numbers of outcome events or summary measures	3-6, Tables 2-3, results

Main results	16	(*a*) Give unadjusted estimates and, if applicable, confounder-adjusted estimates and their precision (eg, 95% confidence interval). Make clear which confounders were adjusted for and why they were included	4-6, results
		(*b*) Report category boundaries when continuous variables were categorized	-
		(*c*) If relevant, consider translating estimates of relative risk into absolute risk for a meaningful time period	-
Other analyses	17	Report other analyses done—eg analyses of subgroups and interactions, and sensitivity analyses	4-6, Tables 2-5, results
**Discussion**			
Key results	18	Summarise key results with reference to study objectives	6,7, discussion
Limitations	19	Discuss limitations of the study, taking into account sources of potential bias or imprecision. Discuss both direction and magnitude of any potential bias	8, discussion
Interpretation	20	Give a cautious overall interpretation of results considering objectives, limitations, multiplicity of analyses, results from similar studies, and other relevant evidence	6-8, discussion
Generalisability	21	Discuss the generalisability (external validity) of the study results	8, discussion
**Other information**			
Funding	22	Give the source of funding and the role of the funders for the present study and, if applicable, for the original study on which the present article is based	11
