# Supplementary material for: The Glycemia Risk Index (GRI) as a Biomarker for Subclinical Endothelial Dysfunction in Type 1 Diabetes: A Cross-Sectional Study
Source: Int J Mol Sci. 2025 Sep 20;26(18):9196. doi: 10.3390/ijms26189196 (PMC12470873; doi:10.3390/ijms26189196)
Supplement: Supplementary file 1 [file ijms-26-09196-s001.zip › Table S1_GRI_R-squared and linear regression equations.pdf]

Table S1. Linear regression analysis between different EPC phenotypes levels (dependent variable) and GRI (independent variable).

	**β**	**P**	R^2	$y=\beta_0+\beta_1x$
$CD34^+$	-1.079	0.006	0.06	y=269.2-1.079x
$CD133^+$	-0.426	0.233	0.01	y=229.8-0.426x
KDR^+	-0.481	0.089	0.02	y=126.4-0.481x
$CD34^+CD133^+$	-0.581	0.008	0.06	y=118.1-0.581x
$CD34^+KDR^+$	-0.147	0.010	0.05	y=24.1-0.147x
$CD133^+KDR^+$	0.020	0.209	0.01	y=4.39-0.020x
$CD34^+CD133^+KDR^+$	0.007	0.437	0.01	y=2.2-0.007x
