# Supplementary material for: The Glycemia Risk Index (GRI) as a Biomarker for Subclinical Endothelial Dysfunction in Type 1 Diabetes: A Cross-Sectional Study
Source: Int J Mol Sci. 2025 Sep 20;26(18):9196. doi: 10.3390/ijms26189196 (PMC12470873; doi:10.3390/ijms26189196)
Supplement: Supplementary file 1 [file ijms-26-09196-s001.zip › Table S2-GRI_Multivariate_Regression_adj.pdf]

Table S2. Multiple linear regression analysis between EPC levels and GRI, in unadjusted and adjusted models.

CD34^{+}	**β [95%IC]**	**P**
Model 1	-1.083 [-1.846; -0.320]	0.006
Model 2	-1.128 [-1.923; -0.334]	0.006
Model 3	-1.094 [-1.883; -0.306]	0.007
Model 4	-1.024 [-1.814; -0.233]	0.012
CD133^{+}		
Model 1	-0.442 [-1.142; 0.258]	0.214
Model 2	-0.408 [-1.137; 0.321]	0.270
Model 3	-0.389 [-1.118; 0.340]	0.293
Model 4	-0.317 [-1.047; 0.413]	0.391
KDR^{+}		
Model 1	-0.460 [-1.001; 0.082]	0.096
Model 2	-0.554 [-1.114; 0.007]	0.053
Model 3	-0.568 [-1.129; -0.007]	0.047
Model 4	-0.501 [-1.060; 0.058]	0.078
CD34^{+}***/CD133***$^{+}$		
Model 1	-0.597 [-1.021; -0.174]	0.006
Model 2	-0.597 [-1.038; -0.156]	0.008
Model 3	-0.579 [-1.017; -0.140]	0.010
Model 4	-0.625 [-1.063; -0.187]	0.006
CD34^{+}***/KDR***$^{+}$		
Model 1	-0.141 [-0.245; -0.037]	0.009
Model 2	-0.166 [-0.274; -0.059]	0.003
Model 3	-0.166 [-0.273; -0.058]	0.003
Model 4	-0.165 [-0.274; -0.056]	0.003
CD133^{+}***/KDR***$^{+}$		
Model 1	0.019 [-0.012; 0.050]	0.224
Model 2	0.015 [-0.017; 0.047]	0.348
Model 3	0.015 [-0.017; 0.047]	0.365
Model 4	0.016 [-0.016; 0.049]	0.324
CD34^{+}***/CD133***$^{+}$***/KDR***$^{+}$		
Model 1	0.007 [-0.011; 0.024]	0.455
Model 2	0.001 [-0.017; 0.019]	0.926
Model 3	0.001 [-0.017; 0.019]	0.909
Model 4	0.001 [-0.017; 0.019]	0.880

Model 1: unadjusted; Model 2: adjusted for age (years); Model 3: adjusted for age (years), diabetes duration (years); Model 4: adjusted for age (years), diabetes duration (years), body mass index (Kg/m^2).
