# Supplementary material for: The Glycemia Risk Index (GRI) as a Biomarker for Subclinical Endothelial Dysfunction in Type 1 Diabetes: A Cross-Sectional Study
Source: Int J Mol Sci. 2025 Sep 20;26(18):9196. doi: 10.3390/ijms26189196 (PMC12470873; doi:10.3390/ijms26189196)
Supplement: Supplementary file 1 [file ijms-26-09196-s001.zip › Table S4-HbA1c_Multivariate_Regression_adj.pdf]

Table S4. Multiple linear regression analysis between EPC levels and HbA_{1c}, in unadjusted and adjusted models.

CD34⁺	**β [95%IC]**	**P**
Model 1	-10.02 [-23.0; 2.95]	0.129
Model 2	-9.93 [-22.97; 3.11]	0.134
Model 3	-11.87 [-24.86; 1.13]	0.073
Model 4	-10.71 [-23.71; 2.29]	0.106
$CD133^+$		
Model 1	7.063 [-4.60; 18,73]	0.233
Model 2	7.282 [-4.42; 18.98]	0.220
Model 3	6.489 [-5.33; 18.31]	0.279
Model 4	7.739 [-4.04; 19.52]	0.196
KDR^+		
Model 1	-5.985 [-15.04; 3.07]	0.193
Model 2	-6.173 [-15.26; 2.91]	0.181
Model 3	-5.681 [-14.87; 3.51]	0.223
Model 4	-4.622 [-13.75; 4.51]	0.318
$CD34^+/CD133^+$		
Model 1	-0.670 [-7.93; 6.59]	0.855
Model 2	0.535 [-7.82; 6.75]	0.885
Model 3	-1.482 [-8.77; 5.81]	0.688
Model 4	-2.070 [-9.38; 5.24]	0.576
$CD34^+/KDR^+$		
Model 1	-1.508 [-3.27; 0.26]	0.093
Model 2	-1.566 [-3.32; 0.21]	0.083
Model 3	-1.636 [-3.42; 0.15]	0.072
Model 4	-1.611 [-3.42; 0.19]	0.080
$CD133^+/KDR^+$		
Model 1	-0.165 [-0.68; 0.35]	0.529
Model 2	-0.181 [-0.70; 0.34]	0.490
Model 3	-0.160 [-0.69; 0.36]	0.546
Model 4	-0.143 [-0.67; 0.39]	0.593
$CD34^+/CD133^+/KDR^+$		
Model 1	-0.144 [-0.43; 0.15]	0.328
Model 2	-0.163 [-0.45; 0.12]	0.259
Model 3	-0.175 [-0.46; 0.11]	0.231
Model 4	-0.173 [-0.46; 0.12]	0.243

Model 1: unadjusted; Model 2: adjusted for age (years); Model 3: adjusted for age (years), diabetes duration (years); Model 4: adjusted for age (years), diabetes duration (years), body mass index (Kg/m^2).
