# Supplementary figures and images for: The Glycemia Risk Index (GRI) as a Biomarker for Subclinical Endothelial Dysfunction in Type 1 Diabetes: A Cross-Sectional Study
Source: Int J Mol Sci. 2025 Sep 20;26(18):9196. doi: 10.3390/ijms26189196 (PMC12470873; doi:10.3390/ijms26189196)

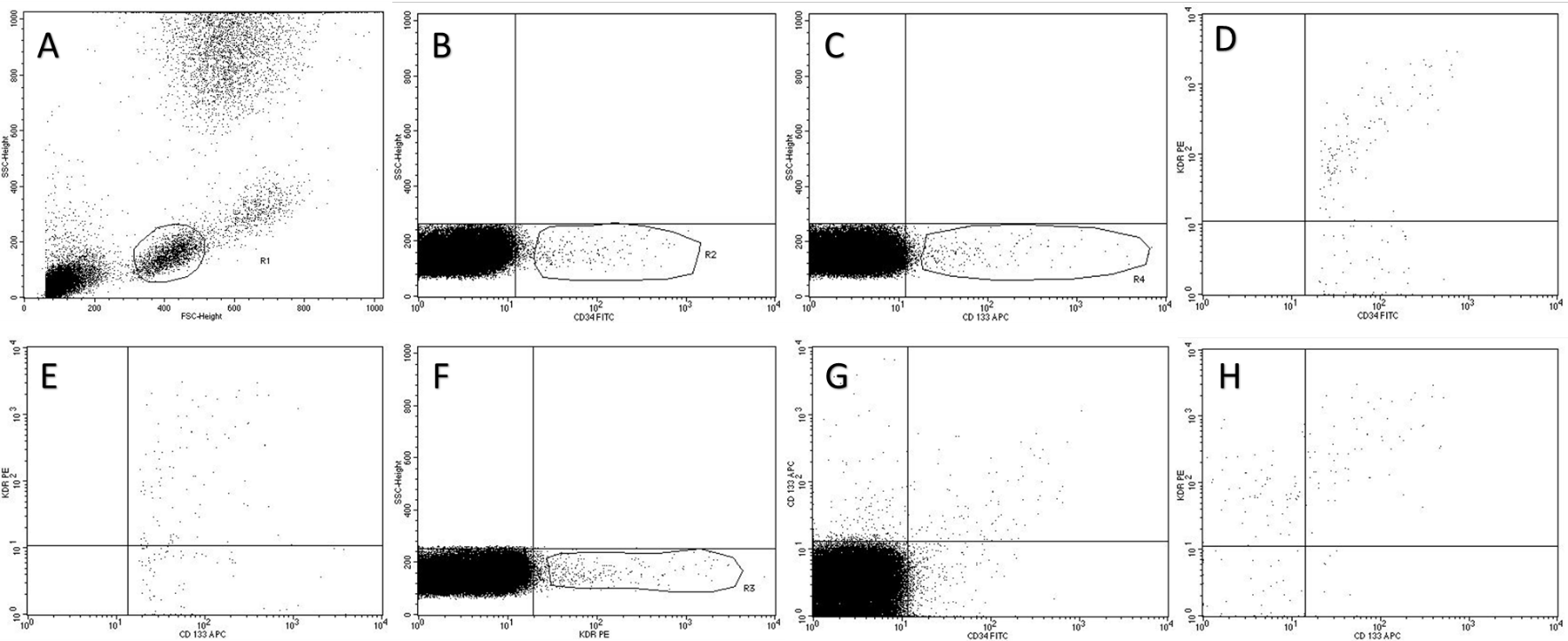

Supplement: Supplementary file 1 [file ijms-26-09196-s001.zip › Figure S1.png]
